# 3D electrogenerated chemiluminescence: from surface-confined reactions to bulk emission

**DOI:** 10.1039/c5sc01530h

**Published:** 2015-06-11

**Authors:** Milica Sentic, Stéphane Arbault, Laurent Bouffier, Dragan Manojlovic, Alexander Kuhn, Neso Sojic

**Affiliations:** a Univ. Bordeaux , Institut des Sciences Moléculaires , CNRS UMR 5255, ENSCBP , 33607 Pessac , France . Email: Alexander.Kuhn@enscbp.fr ; Email: Neso.Sojic@enscbp.fr ; Fax: +33 54000 2717 ; Tel: +33 54000 2496; b Faculty of Chemistry , University of Belgrade , P.O. Box 158 , 11001 Belgrade , Serbia

## Abstract

Among luminescence techniques, electrogenerated chemiluminescence (ECL) provides a unique level of manipulation of the luminescent process by controlling the electrochemical trigger. Despite its attractiveness, ECL is by essence a 2D process where light emission is strictly confined to the electrode surface. To overcome this intrinsic limitation, we added a new spatial dimension to the ECL process by generating 3D ECL at the level of millions of micro-emitters dispersed in solution. Each single object is addressed remotely by bipolar electrochemistry and they generate collectively the luminescence in the bulk. Therefore, the entire volume of the solution produces light. To illustrate the generality of this concept, we extended it to a suspension of multi-walled carbon nanotubes where each one acts as an individual ECL nano-emitter. This approach enables a change of paradigm by switching from a surface-limited process to 3D electrogenerated light emission.

## Abstract 2

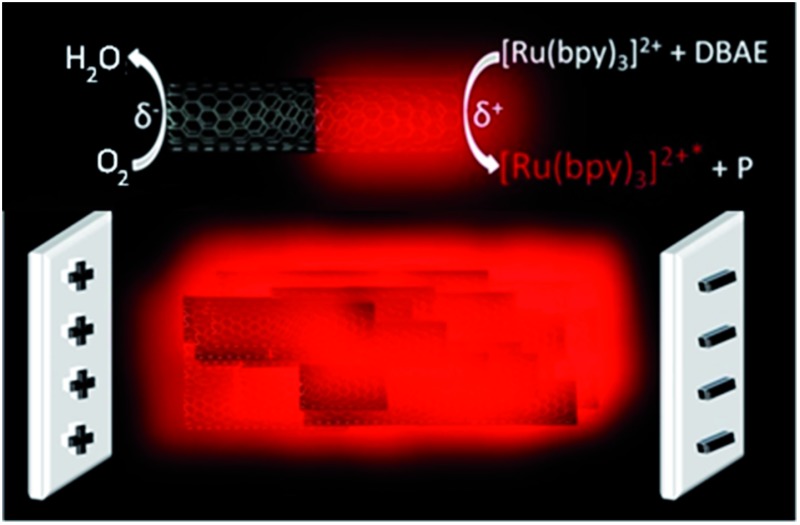
Electrogenerated chemiluminescence is extended to the 3D by generating light at the level of millions of micro-emitters addressed remotely by bipolar electrochemistry.

## Introduction

**Fig. 1:**
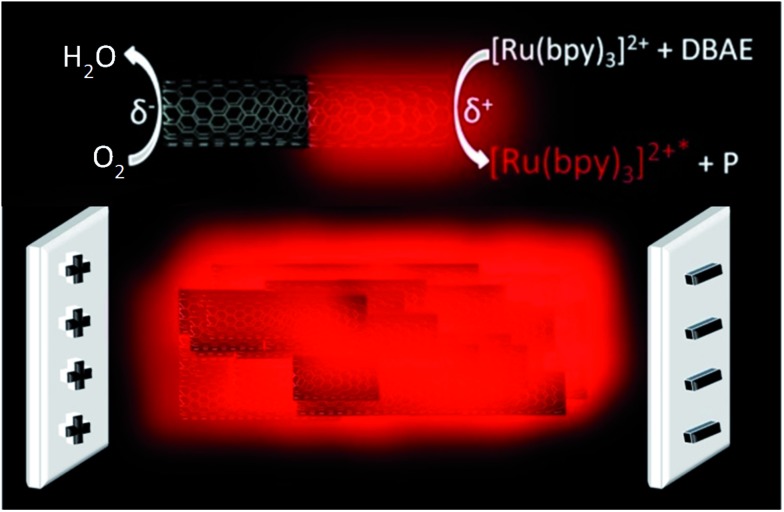
Principle of bulk ECL produced by a CNT suspension. The CNTs are polarized by the electric field generated between the feeder electrodes and redox reactions are triggered at the opposite sides of the object: reduction of oxygen at the cathodic pole (left) and oxidation of the ECL reagents at the anodic pole (right) leading to light emission. Each individual CNT induces ECL emission in a wireless manner so that the entire solution produces a homogeneous ECL light at the macroscopic scale.

Electrogenerated chemiluminescence (ECL) is the phenomenon of light emission by the excited state of a luminophore produced upon an initial electrochemical activation.^1^ The process starts with an electron-transfer reaction at the electrode surface that induces a cascade of reactions leading *in fine* to the excited state. This excited state emits photons during its relaxation to the ground state. ECL has attracted fundamental interest in photochemistry and in electrochemistry because it combines intimately both fields at different steps of the global process.^2–4^ The discovery of ECL emission in aqueous media has led to major applications in analytical chemistry, especially for sensitive immunoassays commercialized for clinical diagnostics.^5–7^ ECL is thus a remarkably versatile method which offers many advantages such as high sensitivity, extremely low background, high linear dynamic range, selectivity, stability of the luminophore, temporal resolution, and easy conjugation of the ECL-label to biomolecules such as antibodies, DNA or RNA. In addition, it does not require any external excitation-light source in contrast to fluorescence. The most common system consists of the luminophore label Ru(bpy)_3_^2+^, or one of its derivatives and a sacrificial co-reactant. However, ECL emission is only generated at the electrode surface or in its immediate vicinity.^8–11^ Indeed, on one hand, direct electron-transfer reactions are spatially limited to nanometric distances from the electrode surface. On the other hand, considering the different mechanistic pathways with diffusing co-reactant radicals, ECL may occur only in the nanometric to micrometric regions close to the electrode surface.^8–11^ Therefore, ECL is by nature a 2D process, which is strictly confined to the geometric surface area of the electrode. This appears, so far, as an intrinsic limitation compared, for example, to chemiluminescence where reagents are mixed and react homogeneously to form the excited state in the bulk.^12^ Investigating original designs and concepts to overcome this inherent barrier due to the initial electrochemical step is therefore of paramount importance for fundamental research and applied detection strategies, for example, in bioanalytical assays. Here, for the first time, we demonstrate experimentally ECL generation in a 3D format based on an original approach (Fig. 1) using a dispersion of conductive micro- or nano-objects that are remotely addressed by bipolar electrochemistry (BPE).

BPE is a powerful wireless method which promotes electrochemical reactions at the extremities of conductive objects placed in solution when applying an electric field.^13,14^ The potential difference between the object and the solution varies across its length due to the electric field. This results in a polarization voltage occurring between both extremities of the object. If the polarization is high enough, asymmetric redox reactivity can be carried out at the opposite extremities of the conductive particle: oxidation reactions at the anodic pole, simultaneously with reduction reactions at the cathodic pole. It is therefore a wireless approach where electrochemical reactions are induced at both poles without a direct physical contact between the polarized object and the electrodes generating the electric field. BPE is an important method for a wide variety of applications, from analysis to materials science.^15–21^ For example, Crooks *et al.* reported ECL emission at the level of 1000 bipolar microelectrodes fabricated on a glass slide.^22^ But, even in this case, ECL is still confined to the surface of the microelectrode array. We exploited recently the versatility of BPE and its wireless characteristics to develop ECL-emitting bioelectrochemical swimmers.^23,24^ Indeed, BPE induces simultaneously the production of gas bubbles for propelling the swimmers and enzymatic formation of the activated ECL reagents, which generate light only in the presence of glucose. Due to the wireless capabilities of BPE, single or thousands of conductive objects can be simultaneously addressed by this versatile technique.^13,22^ Moreover, there is no topological limitation, so the bipolar electrodes can have micro- or nano-scale dimensions.^25^ Herein, through a rational choice of model ECL systems and a simple design of the BPE setup, we demonstrate the efficient generation of bulk ECL (*i.e.* 3D ECL) at the level of a dispersion of conductive micro- or nano-objects in a capillary. Each single object is addressed electrochemically by BPE and generates ECL light. Indeed, each microbead or multi-walled carbon nanotube (MWCNT) is polarized by the electric field and ECL emission is therefore triggered in a wireless manner simultaneously on all objects. Thus the entire solution emits ECL light, which can be considered as a change of paradigm with respect to the classic 2D approach. Eventually, the reported approach may avoid all the delicate immobilization steps on the electrode surface and allows switching from heterogeneous diffusion-limited reactions to homogeneous reactions with much higher kinetic rates and improved analytical performances.

## Results and discussion

Fig. 1 displays the simple design of the 3D ECL setup. It comprises a suspension of conductive objects placed in a capillary between 2 feeder electrodes. As a proof-of-principle, we demonstrated this concept in a first set of experiments with glassy carbon beads and then extended it to a suspension of MWCNTs. The feeder electrodes serve to apply the electric field *E* which polarizes the conductive objects. Eqn (1) shows that the polarization voltage Δ*V* induced between both extremities of the object is proportional to the external electric field *E* and to the characteristic dimension *l* of the object:^14^

1 Δ*V* = *El*

When a sufficient polarization voltage Δ*V* is induced, each object acts as a bipolar electrode and redox reactions occur cooperatively at both extremities: oxidation of the ECL reagents at the anodic pole of the object and reduction of either dissolved oxygen or of H_2_O at the cathodic pole (Fig. 1). Because of charge neutrality during BPE, reduction occurs simultaneously at the cathodic pole with an equal intensity.

**Fig. 2:**
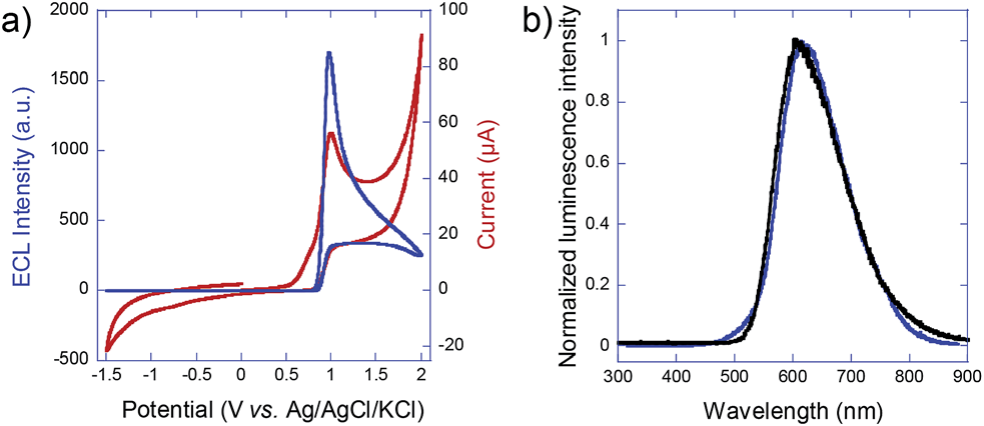
(a) Cyclic voltammogram (red curve) and ECL signal (blue curve) of 2.5 mM Ru(bpy)_3_^2+^ in the presence of 20 mM DBAE in PBS buffer (pH 7.4) recorded on a home-made glassy carbon electrode at a scan rate of 50 mV s^–1^. (b) Comparison of the ECL spectrum, generated by the beads (blue curve), and the photoluminescence spectrum of Ru(bpy)_3_^2+^ (black curve).

To estimate the required polarization, we recorded both voltammetric and ECL signals at a conventional working electrode made out of the glassy carbon bead material (Fig. 2a). ECL light (blue curve) is generated at +1.0 V *vs.* Ag/AgCl and the cathodic current (red curve) starts to increase significantly at –1.4 V *vs.* Ag/AgCl, so that a minimal polarization Δ*V* of 2.4 V is required for coupling both reactions in the bipolar configuration. In the first set of experiments with carbon beads having an average diameter of 30 μm, eqn (1) states that the corresponding field has to be higher than 0.8 kV cm^–1^, in a first-order approximation. The carbon beads were dispersed in an agarose gel inside a capillary. The feeder electrodes were placed just outside of the capillary (see Fig. 5 in the Experimental section). The ECL experiments were performed in a buffer solution containing the model system Ru(bpy)_3_^2+^ and 2-(dibutylamino)ethanol (DBAE) as the luminophore and the co-reactant, respectively.^26^

**Fig. 3:**
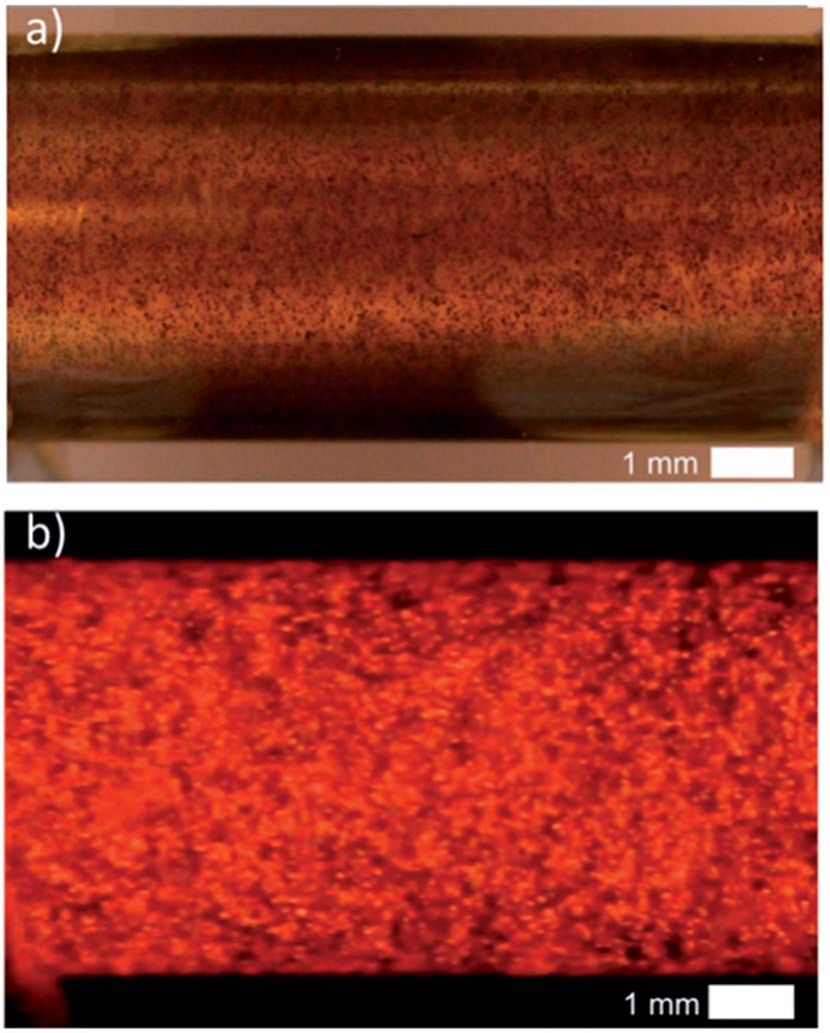
3D ECL emission by carbon beads immobilized in a capillary. (a) White light image of the glassy carbon beads (average radius: 15 μm; concentration: approx. 300 000 per mL) immobilized in an agarose gel inside a capillary. (b) Image of the ECL emitted by the carbon beads polarized by BPE. The capillary contained 1.4% agarose gel in 4 mM PBS buffer with 2.5 mM Ru(bpy)_3_^2+^ and 20 mM DBAE. The applied electric field was 1 kV cm^–1^.

Fig. 3a shows a white-light image of the glassy carbon beads inside the capillary. Each dark dot corresponds to a single carbon bead. Agarose gel was used to avoid the sedimentation of the particles and to keep them well-separated during experiments. The ambient light was then turned off and an electric field of 1 kV cm^–1^ was applied. This value is slightly higher than the previously calculated theoretical threshold value in order to polarize also the beads which are slightly smaller than the average size. ECL emission was extremely bright and it could be observed with the naked eye or recorded with a smartphone camera. ECL intensity was so strong that it was easily seen even under ambient light. The ECL image presented in Fig. 3b was indeed recorded with a consumer digital camera. One can observe that the entire content of the capillary emits ECL. Each bead is polarized remotely and ECL is generated at the anodic pole of each micro-object. Even though discrete spots of ECL light could be distinguished at the sub-millimeter scale, the very large number of emitters allows the generation of a very homogeneous ECL signal at a macroscopic scale. Indeed, ECL was relatively uniform, due to the spatial distribution of the ECL emitters (*i.e.* the carbon beads in this first example).

To prove that the emitted light corresponds to an ECL phenomenon, the spectrum of the 3D emission was recorded and compared to the well-known photoluminescence of the Ru(bpy)_3_^2+^ luminophore. As shown in Fig. 2b, emission spectra are identical with both maxima peaking at 610 nm, thus indicating that the same excited state is generated upon bipolar electrochemical and photochemical excitation. This is a clear demonstration of the ECL nature of the 3D emission triggered by BPE.

CNTs are particularly fascinating objects with strong anisotropy. Indeed, the diameter of a single MWCNT is a few nanometers, while its length may be in the micro- or even millimeter range. Moreover, their conductivity is high along the CNT axis.^27^ Both features make them a particularly attractive material for BPE applications. As already mentioned, the electric field required to polarize an object with a given amplitude is inversely proportional to its characteristic length. In other words, much higher electric fields are necessary for nanometric particles compared to micrometric ones. Uniform and stable suspensions of CNTs may be prepared by simple procedures using, for example, adsorption of surfactants on their surface.^28^ From a BPE point of view, such CNT suspensions represent a dispersion of potential bipolar electrodes. Warakulwit *et al.* have shown that CNTs dispersed in solution may be electrochemically modified by a gold deposit formed selectively at its cathodic side by BPE.^25^ Thus, the concept of 3D ECL may be possibly extended to the nanoscale by exploiting such remarkable properties of CNT suspensions.

**Fig. 4:**
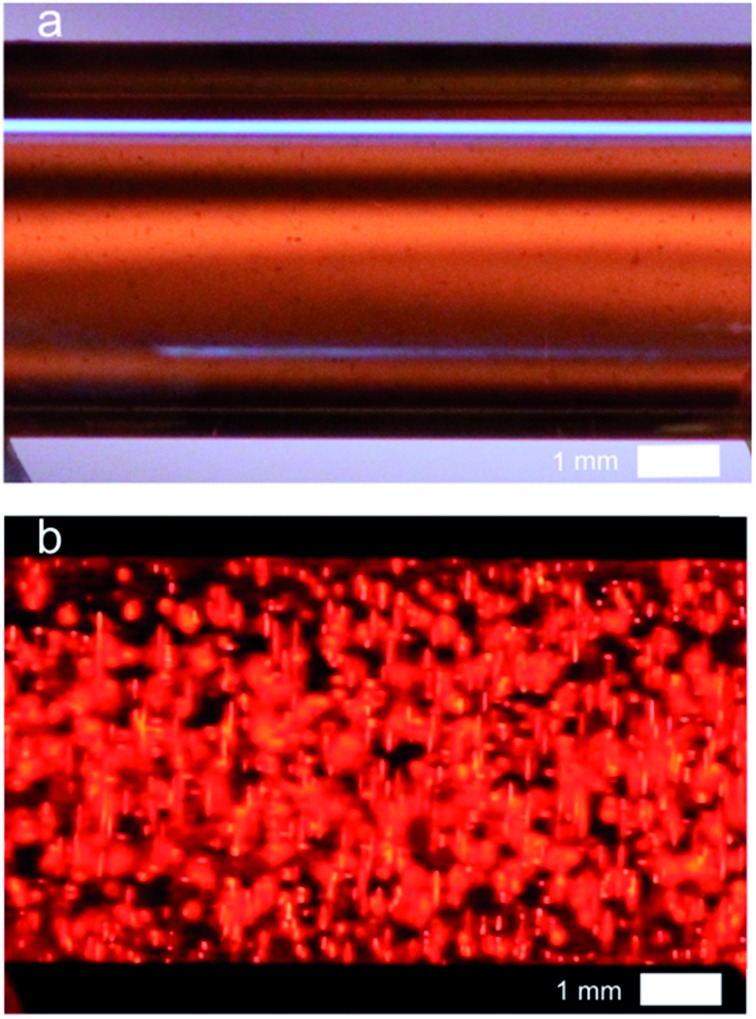
3D ECL emission at the level of a MWCNT suspension in a capillary. (a) Image of the suspension of CNTs under white light before applying the electric field. The capillary is filled with 4 mM PBS containing 2.5 mM Ru(bpy)_3_^2+^, 1.4% of agarose, 20 mM DBAE and the MWCNTs. A 0.5 μL suspension of SDS suspended MWCNTs (0.005% wt) was diluted in 0.5 mL of the solution containing the ECL reagents and injected in the capillary. (b) Image in the dark of the bulk ECL emitted by the CNTs. Applied external electric field: 1.2 kV cm^–1^.

Due to the polarization constraints inherent to BPE, we selected relatively long MWCNTs with an average length of 20 μm. Such dimensions allow addressing them electrochemically with an electric field only slightly higher (1.2 kV cm^–1^) than the one used for the microbeads. The MWCNTs were first dispersed in the capillary as illustrated by the white-light image (Fig. 4a). In the dark, when applying the electric field strong ECL light is emitted and recorded in the whole capillary (Fig. 4b). Indeed, each MWCNT is polarized and oxidation of Ru(bpy)_3_^2+^ and of DBAE occurs at the anodic extremity, resulting in ECL emission. ECL is generated by single MWCNTs and probably also by aggregates that are not perfectly dispersed (Fig. 4b). In comparison to the beads, the size of each emitter is decreased, but the overall quantity of bipolar electrodes and thus the number of ECL emitters is increased. Therefore the entire volume of the solution produces luminescence thus leading to quasi-bulk (*i.e.* 3D) ECL emission.

## Conclusions

In this contribution, we demonstrate for the first time the bulk generation of ECL in a 3D configuration. This has been possible by using BPE as a straightforward approach to address electrochemically millions of micro- or nano-objects simultaneously in a wireless way. Every object acts as an individual emitter of an electrochemically generated luminescence signal and their collective behavior enables strong light emission in the whole volume of the solution. This represents a very significant advantage with respect to classic ECL configurations, where the emission is intrinsically confined to the close vicinity of macroscopic electrodes, thus limiting its intensity and visibility. It can therefore be expected that such a bulk emission of light will open the door for a whole range of new applications of ECL such as high-sensitivity analysis or optical tracking of nanomotors.

## Experimental

### Materials

All chemicals were of analytical reagent grade and were used as received. Solutions were prepared using Milli-Q water (resistivity = 18 MΩ cm). Tris(2,2′-bipyridyl) dichlororuthenium(ii) hexahydrate, sodium phosphate dibasic heptahydrate, sodium phosphate monobasic monohydrate, agarose, sodium dodecyl sulfate (SDS) and 2-(dibutylamino)ethanol (DBAE) were purchased from Sigma-Aldrich. Glassy carbon beads were purchased from Alfa Aesar. Multi-walled carbon nanotubes (MWCNTs) were provided by Philippe Poulin (CRPP).

### Method

**Fig. 5:**
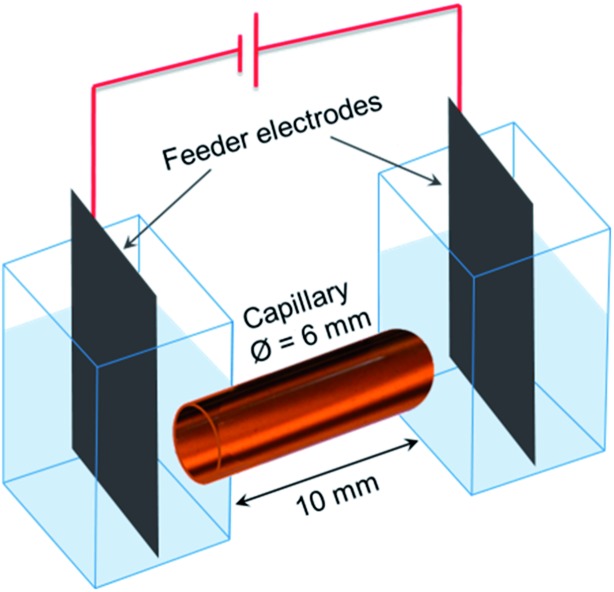
Scheme of the set-up used for 3D ECL experiments. The capillary is filled with a suspension of the conducting objects (*i.e.* carbon microbeads or MWCNTs) and the ECL reagents. Both compartments that contain the graphite feeder electrodes are connected by the capillary.

A home-made spectroelectrochemical cell (Fig. 5) was used for the 3D ECL generation. Two compartments containing the feeder electrodes (graphite sheets) were connected with a glass capillary (outer diameter: 6 mm; length: 10 mm). The cell was filled with a PBS solution (pH 7.4) containing 2.5 mM tris(2,2′-bipyridyl) dichlororuthenium(ii) hexahydrate, 1.4% of agarose, 20 mM DBAE and the conductive objects. Agarose was used to avoid the sedimentation of the bipolar electrodes and to fill easily the capillary. We generated 3D ECL in a first set of experiments with glassy carbon beads (approx. 300 000 beads per mL) and then with a dispersion of MWCNTs. 0.5 μL of SDS-suspended MWCNTs (0.005% wt) were diluted in 0.5 mL of the solution containing the ECL reagents and injected in the capillary. The filled capillary is very stable and it can be prepared a few minutes or hours before generating 3D ECL. The electric field was applied using a DC generator (Heinzinger 10000-200 pos). The white-light and ECL pictures were recorded using an SLR commercial digital camera (Canon 60 D; Raw mode imaging in full frame resolution) equipped with a lens for macrophotography (Canon 100 mm-f:2.8).

### Electrochemical and ECL characterization

Cyclic voltammetry experiments were performed with μ-Autolab Type III and PGSTAT30 electrochemical stations. Electrochemical measurement was combined with a simultaneous monitoring of the ECL intensity by using a Hamamatsu photomultiplier tube R5070A. The three-electrode system consisted of a home-made glassy carbon electrode as working electrode, composed of the same material as the beads used in the 3D ECL experiments, a Ag/AgCl/KCl 3 M reference electrode and a platinum wire counter-electrode. ECL spectra were acquired with a Princeton Instruments Model Acton SpectraPro 2300i system equipped with a CCD camera.
